# Comprehensive analyses of tumor immunity: implications for cancer immunotherapy

**DOI:** 10.1186/s13059-016-1028-7

**Published:** 2016-08-22

**Authors:** Bo Li, Eric Severson, Jean-Christophe Pignon, Haoquan Zhao, Taiwen Li, Jesse Novak, Peng Jiang, Hui Shen, Jon C. Aster, Scott Rodig, Sabina Signoretti, Jun S. Liu, X. Shirley Liu

**Affiliations:** 1 Department of Biostatistics and Computational Biology, Dana Farber Cancer Institute, 450 Brookline Ave., Boston, MA 02215 USA; 2 Department of Statistics, Harvard University, 1 Oxford St., Cambridge, MA 02138 USA; 3 Department of Pathology, Brigham and Women’s Hospital and Harvard Medical School, 75 Francis St., Boston, MA 02215 USA; 4 State Key Laboratory of Oral Diseases, West China Hospital of Stomatology, Sichuan University, 14 Renmin South Rd 3rd Section, Wuhou, Chengdu, Sichuan 610041 China; 5 Center for Epigenetics, Van Andel Research Institute, 333 Bostwick Ave N.E., Grand Rapids, MI 49503 USA

**Keywords:** Cancer immunity, Tumor immune infiltration, Cancer immunotherapies, Cancer vaccine, Checkpoint blockade

## Abstract

**Background:**

Understanding the interactions between tumor and the host immune system is critical to finding prognostic biomarkers, reducing drug resistance, and developing new therapies. Novel computational methods are needed to estimate tumor-infiltrating immune cells and understand tumor–immune interactions in cancers.

**Results:**

We analyze tumor-infiltrating immune cells in over 10,000 RNA-seq samples across 23 cancer types from The Cancer Genome Atlas (TCGA). Our computationally inferred immune infiltrates associate much more strongly with patient clinical features, viral infection status, and cancer genetic alterations than other computational approaches. Analysis of cancer/testis antigen expression and CD8 T-cell abundance suggests that MAGEA3 is a potential immune target in melanoma, but not in non-small cell lung cancer, and implicates SPAG5 as an alternative cancer vaccine target in multiple cancers. We find that melanomas expressing high levels of CTLA4 separate into two distinct groups with respect to CD8 T-cell infiltration, which might influence clinical responses to anti-CTLA4 agents. We observe similar dichotomy of TIM3 expression with respect to CD8 T cells in kidney cancer and validate it experimentally. The abundance of immune infiltration, together with our downstream analyses and findings, are accessible through TIMER, a public resource at http://cistrome.org/TIMER.

**Conclusions:**

We develop a computational approach to study tumor-infiltrating immune cells and their interactions with cancer cells. Our resource of immune-infiltrate levels, clinical associations, as well as predicted therapeutic markers may inform effective cancer vaccine and checkpoint blockade therapies.

**Electronic supplementary material:**

The online version of this article (doi:10.1186/s13059-016-1028-7) contains supplementary material, which is available to authorized users.

## Background

Cancer immunotherapy has recently achieved remarkable success in treating late stage tumors [1, 2], but a substantial fraction of patients failed to respond [3, 4]. Efforts have been made to elucidate the tumor–immune interactions [5–8] and provide prognostic predictors [9–11]. Rooney et al. [6] studied cytolytic activity (CYT) using the expression levels of two effector molecules and identified possible mechanisms of immune evasion. Another recent work [7] characterized the immunophenotypes in colorectal cancer and provided novel therapeutic targets. While these studies profoundly improved the understanding of cancer immunoediting [12], less is known about how the interactions between tumor and the immune system impact patient outcome. Clinical investigations on tumor-infiltrating immune cells have established the roles of cytotoxic T cells (CTLs) and tumor-associated macrophages (TAMs) in some diseases [13, 14]. However, the clinical impact of other immune cells in many cancers remains poorly understood. Hence, there is a great need for a more comprehensive and translational analysis of tumor immunity to better understand the multi-component antitumor response and guide effective immunotherapies in different cancers.

In this work, we integrated the molecular profiles of over 10,000 tumor samples across 23 cancer types to investigate the impact of individual immune components on a wide spectrum of clinical features. Our estimates of tumor-infiltrating immune cells were validated using multiple approaches, including in silico simulation, comparison with orthogonal inferences, and a pathological approach. Correlating immune infiltration with patient outcomes, we identified a number of associations, including both novel associations and those supported by prior studies [14]. Our analysis also suggested that the inter-tumor heterogeneity of immune infiltration is potentially caused by both cancer genetic variations as well as the disease-specific expression pattern of the chemokine/receptor network. As a translational approach, we investigated immunotherapy targets for both therapeutic cancer vaccine and checkpoint blockade. Finally, our in silico inferences and associated findings have been packaged into a web-accessible resource, TIMER (Tumor IMmune Estimation Resource), to enable further explorations of the disease-specific clinical impact of different immune infiltrates in the tumor microenvironment.

## Results

### Computational estimation of tumor immune infiltration

**Fig. 1:**
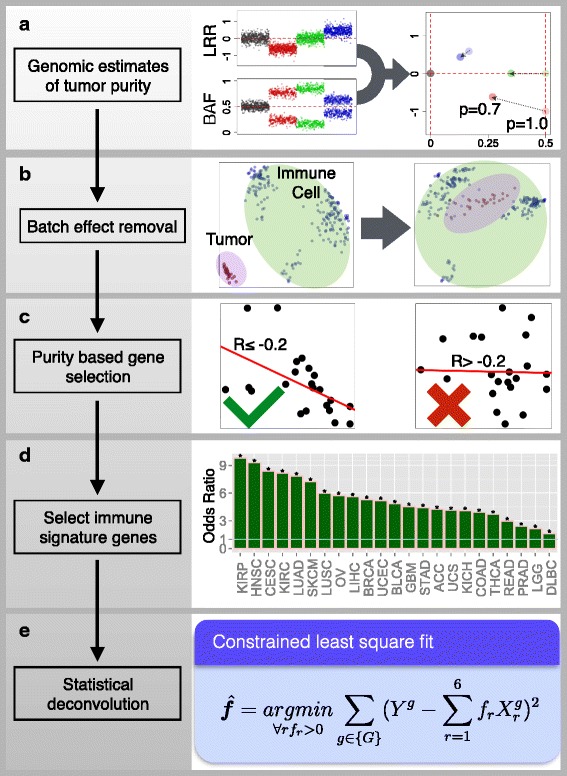
Computational method for estimating the abundance of tumor-infiltrating immune cells. Tumor purity was estimated for each sample using DNA single-nucleotide polymorphism (SNP) array data (**a**). B allele frequency (*BAF*) is the frequency of a randomly selected parental allele. The logR ratio (*LRR*) is the log_2_(Y/2), Y being the marker intensity in the SNP array. TCGA gene expression profiles were combined with reference immune cell expression data after batch effect removal (**b**). Informative genes with expression levels inversely correlated with tumor purity (Pearson’s r ≤ −0.2 and *P* value ≤ 0.05) are selected (**c**) and tested for immune signature enrichment (Fisher’s exact test) (**d**). In all 23 cancers informative genes are significantly enriched for immune signature. Diffuse large B-cell lymphoma (DLBC) has the lowest enrichment (odds ratio = 1.6, q = 0.0005, Fisher’s exact test). In this study, we estimate the abundance of six immune cell types (B cells, CD4 T cells, CD8 T cells, neutrophil, macrophage, and dendritic cells) using selected immune signature genes through constrained least squares fitting (**e**). *Asterisks* in **d** indicate event significance at a 1 % false discovery rate

We developed a computational method to estimate the abundance of six tumor-infiltrating immune cell types (B cells, CD4 T cells, CD8 T cells, neutrophils, macrophages, and dendritic cells) to study 23 cancer types in The Cancer Genome Atlas (TCGA): adenocortical carcinoma (ACC), bladder carcinoma (BLCA), breast carcinoma (BRCA), cervical squamous carcinoma (CESC), colon adenocarcinoma (COAD), diffusive large B-cell lymphoma (DLBC), glioblastoma multiforme (GBM), head and neck carcinoma (HNSC), kidney chromophobe (KICH), kidney renal clear cell carcinoma (KIRC), kidney renal papillary cell carcinoma (KIRP), lower grade glioma (LGG), liver hepatocellular carcinoma (LIHC), lung adenocarcinoma (LUAD), lung squamous carcinoma (LUSC), ovarian serous cystadenocarcinoma (OV), prostate adenocarcinoma (PRAD), rectum adenocarcinoma (READ), skin cutaneous melanoma (SKCM), stomach adenocarcinoma (STAD), thyroid carcinoma (THCA), uterine corpus endometrial carcinoma (UCEC), and uterine carsinosarcoma (UCS). These six immune cell types include some of the currently most promising cancer immunotherapy targets and also have sufficient numbers of reference immune cell samples to make accurate inferences (“Methods”). The prerequisite data include tumor purity, tumor gene expression profiles (as transcript per million reads (TPM)) from TCGA, and an external reference dataset of purified immune cells. Tumor purity is critical to selecting genes informative for deconvolving immune cells in the tumor tissue and was inferred from copy number alteration data using an R package, CHAT, we have developed [15] (Fig. 1a). Our purity estimation method has been validated using diluted series with known tumor/normal mixture ratios and agreed with previous methods and orthogonal estimations [16]. The distributions of tumor purity showed large variations among different samples across most of the 23 TCGA cancer types (Additional file 1: Figure S1). For each cancer dataset, batch effects between TCGA and the external reference data sets were removed using ComBat [17] (Fig. 1b). Next we selected genes whose expression levels are negatively correlated with tumor purity (Fig. 1c; Additional file 1: Figure S2; Additional file 2: Table S1), an indication that these genes are expressed by stromal cells in the tumor microenvironment. Across all 23 cancers, informative genes selected from the above steps are significantly enriched for a predefined immune signature [18] (Fig. 1d). This result indicates that large numbers of immune cell-specific genes are highly expressed in the tumor microenvironment. Finally, we used constrained least squares fitting [19] on the informative immune signature genes to infer the abundance of the six immune cell types (Fig. 1e).

As a key component of TIMER, the outcome of the above method was validated with multiple approaches. The first one was pathology, where we estimated the levels (low, median, and high) of neutrophils in bladder cancer samples using hematoxylin and eosin stained slides from TCGA (“Methods”). Our in silico predictions of neutrophil abundance agreed well with the histological estimations (Additional file 1: Figure S3a, b). We also validated our predictions using total infiltrating leukocytes estimated from DNA methylation data [20] and observed high concordance between our RNA and the DNA-based predictions in all available cancers (Additional file 1: Figure S3c). In addition, Monte Carlo simulations with known immune cell fractions were applied to all cancer types. High correlations were observed between the predicted and simulated immune cell abundance for all comparisons except CD4, CD8 T cells, dendritic cells in GBM, and B cells in DLBC (Additional file 1: Figure S3d; “Methods”), which were excluded from downstream analysis. The inferred relative fractions of the six immune cell types of all the samples across 23 cancers are available in Additional file 3: Table S2.

### Clinical relevance of tumor immune infiltration

**Fig. 2:**
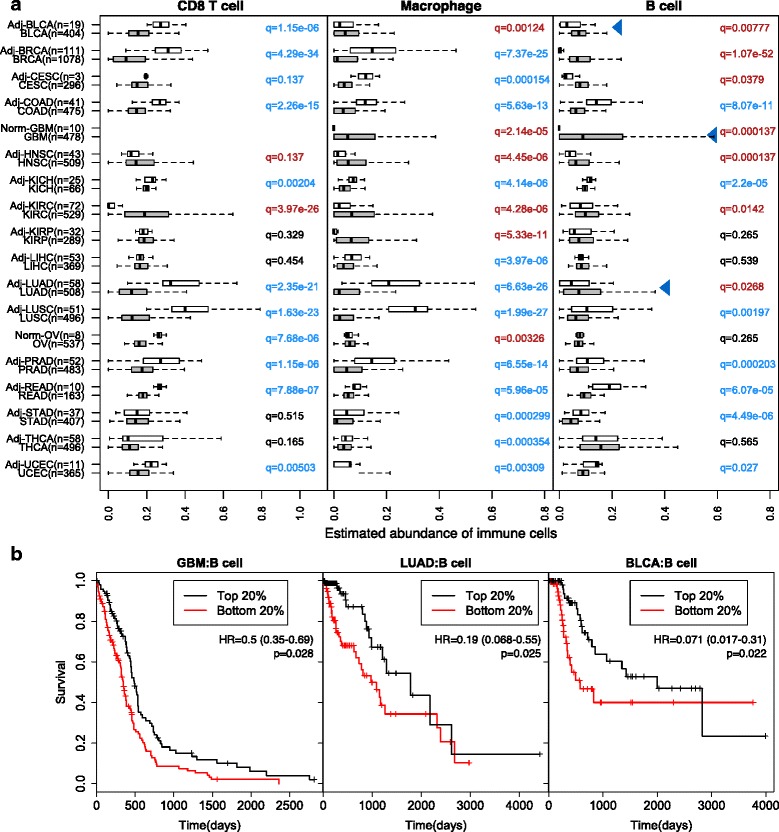
Distribution of infiltrating immune cells and selective enrichment of B cells in the tumor microenvironment. **a** The abundance of infiltrating CD8 T cells, macrophages, and B cells in 18 cancer types, with both primary tumor and adjacent (*Adj*)/normal (*Norm*) tissue available. Normal tissue was from healthy donors where adjacent tissues were unavailable. Statistical significance was evaluated by Wilcoxon rank sum test. *Blue arrowheads* point to three cancers with B cells significantly enriched in the primary tumor and associated with clinical outcomes. q values are colored *red*, *blue*, or *black* for significant (false discovery rate ≤ 0.15) enrichment in tumor, adjacent or normal tissue, or insignificance, respectively. **b** B-cell infiltration level significantly predicted patient survival in selected cancer types. Tumors in the top 20th percentile of B-cell infiltration were compared with those in the bottom 20th percentile. The median survival time for the top 20 % of patients with brain, lung, and bladder cancers was 460, 1778, and 2000 days, respectively, and for the bottom 20 % 345, 976, and 575 days, respectively. Statistical significance and hazard ratios (*HR*) with 95 % confidence intervals were calculated for all the samples, not just the top and bottom 20 %, using multivariate Cox regression including all six immune cell types, patient age, and clinical stage

To study the distribution of infiltrating immune cells in the tumor and adjacent/normal tissues, we focused on 18 cancer types for which the mRNA expression profiles of adjacent or normal tissues were available. Consistent with Rooney et al. [6], CD8 T cells are enriched in tumor tissues in kidney cancer (KIRC) and head and neck cancer. In contrast, CD8 T cells appear to be in lower abundance in most other cancers, such as non-small cell lung carcinomas (including adenocarcinoma and squamous cell carcinoma) and colorectal cancer (including colon and rectal adenocarcinoma) (Fig. 2a). Macrophages are significantly enriched in GBM, which is supported by previous observations showing that microglia and macrophages are present in large numbers in the glioma microenvironment [21]. Furthermore, the abundance of tumor-infiltrating B cells is significantly higher than in the adjacent or normal tissues in eight cancer types. Interestingly, B-cell infiltration predicts a significantly better outcome in a subset of these cancers (Fig. 2b). The most dramatic case is GBM, where patients with the top 20 % of B-cell infiltration have a 4.7-month longer median survival time than those with the lowest 20 % (“Methods”). This result suggests that tumor-infiltrating B cells play an important role in the antitumor responses in GBM and lung and bladder cancers.

**Fig. 3:**
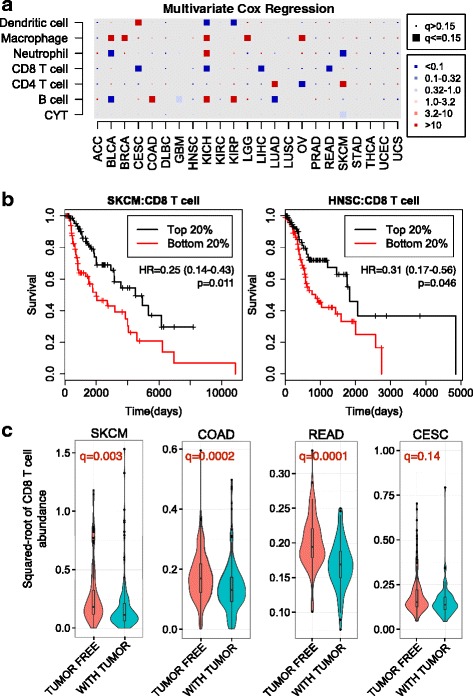
Immune cell infiltration predicts clinical outcome. **a** Association of tumor infiltrating immune cells with patient survival. For each cancer type, multivariate Cox regression was performed, with covariates including the abundance of six immune cell types, patient age at diagnosis, clinical stage, and viral infection status when available. Each entry on the first six rows of the heatmap represents the hazard ratio (*HR*) of a corresponding immune cell type, with larger size indicating statistical significance at a false discovery rate (FDR) of 0.15 and color indicating the value of the HR. The last row of the heatmap records the Cox model HRs and statistical significance using cytolytic activity (*CYT*) scores adjusted for the same covariates. Multiple test correction was performed using q value across cancer types and six immune components. **b** Kaplan–Meier curves of melanoma (SKCM) and head and neck cancer (HNSC) stratified by infiltration CD8 T-cell abundance. Median survival time for the top 20 % of patients in melanoma and head and neck cancers is 4507 and 1838 days, respectively, and for bottom 20 % 2005 and 862 days, respectively. Statistical significance, hazard ratios, and 95 % confidence intervals were calculated using multivariate Cox regression and all the samples as described above. **c** CD8 T-cell infiltration in primary tumors (metastatic samples for SKCM) significantly (FDR ≤ 0.15) predicts tumor relapse in selected cancers. Statistical significance was evaluated using logistic regression correcting for patient age and clinical stage

We next investigated how infiltrating immune cells influence clinical outcomes. Using multivariate Cox regression adjusted for age, stage, and viral infection status, we identified many significant associations between immune cell abundance and patient outcomes (Fig. 3). While CD8 T-cell infiltration associates with prolonged survival and macrophage infiltration consistently predicts worse outcome, other immune cell types have cancer-specific effects on prognosis (Fig. 3a). These observations corroborate previous reports that cytotoxic T-cell infiltration independently predicts better outcome in liver [22] and rectal [23] cancers. Consistent with established knowledge in melanoma and head and neck cancer [24, 25], we also found infiltrating CD8 T cells to be associated with survival in univariate analysis (Fig. 3b). However, it is not an independent predictor of better outcome after adjusting for other covariates, since CD8 T cells are correlated with neutrophil infiltration in melanoma and human papilloma virus (HPV) infection in head and neck cancer (“Methods”). In fact, infiltrations of B cells and CD4 and CD8 T cells are all significantly higher in HPV-positive than in HPV-negative head and neck tumors (Additional file 1: Figure S4), suggesting that viral antigens result in an elevated lymphocyte response. Besides the effect on patient survival, CD8 T cells may also play an important role in preventing tumor recurrence (Fig. 3c). In melanoma and colorectal and cervical cancers, patients with higher CD8 T-cell infiltration in the primary tumors have a significantly lower risk of developing a second tumor during the follow-up period. Overall, our observations on tumor-infiltrating CD8 T cells are extensively supported by clinical studies [14] (Additional file 4: Table S3), thus providing additional validation to our deconvolution method. Cytolytic activity (CYT) is a previously defined metric of immune-mediated cell destruction [6]. Compared with CYT, our analysis identified many more strong associations between tumor-infiltrating immune cells and patient clinical outcomes (Fig. 3a), presumably because our method takes into account expression data from hundreds of genes instead of only two.

TAM numbers have been reported to be a predictor of worse outcome in many cancers [13]. Consistently, we found that TAM significantly associates with worse outcome in bladder, breast, and ovarian cancers and in lower-grade glioma (Fig. 3a), supporting TAMs as an independent prognostic factor for these cancers. Extending this analysis to the less well-studied chromophobe renal carcinoma (KICH), we detected a significant inverse association between macrophage infiltration and patient survival (Fig. 3a), suggesting that TAMs function in KICH as in other solid tumors.

### Potential causes for immune infiltration heterogeneity

**Fig. 4:**
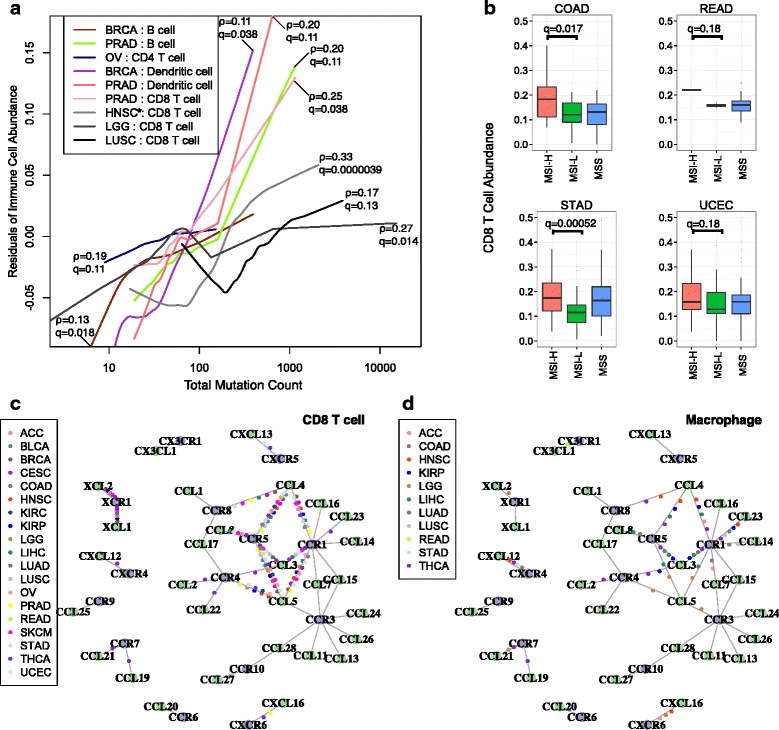
Potential causes of inter-tumor immune infiltration heterogeneity. **a** In selected cancer types, counts of total somatic coding mutations positively associated with the level of infiltrating immune cells. The *y-axis* is the residual of corresponding immune cell abundance after linear regression against tumor purity. Statistical significance was evaluated using partial Spearman’s correlation adjusted for tumor purity. The *asterisk* indicates only HPV-negative tumors were selected for head and neck cancer. Multiple test correction was performed across cancer types and six immune components. *Gray hues* indicate previously known results (HNSC, LGG, and LUSC), while other findings are novel in this study. **b** CD8 T-cell infiltration is associated with microsatellite instability (*MSI*) status in cancers commonly with MSI. *MSI-H* high level of microsatellite instability, *MSI-L* low level of microsatellite instability; *MSS* microsatellite stable. Statistical significance was evaluated using a Wilcoxon rank sum test. **c**, **d** Chemokine/receptor networks for immune infiltration in diverse cancer types. *Vertexes* are ligands (*green*) and receptors (*purple*) and *edges* indicate known molecular interactions. For each cancer, the partial correlations (corrected for purity) between the chemokine gene expression and infiltration of CD8 T cells (**c**) or macrophages (**d**) were calculated. For a pair of interacting chemokine and receptor genes, if both are significantly correlated with immune cell infiltration in one cancer, a *colored dot* represents the cancer type is placed on the edge connecting the chemokine and receptor. Statistical significance was evaluated using partial Spearman’s correlation at a false discovery rate threshold of 0.01. Heatmap visualizations of the same results are shown in Additional file 1: Figure S6c, d

We next examined the possible causes of inter-tumor immune infiltration heterogeneity, such as somatic mutations and chemokine expression. Non-synonymous somatic mutations in the tumor genome can generate immunogenic neoantigens that trigger antitumor response through T-cell activation [26–29]. In addition, total mutation load has been suggested as a surrogate for neoantigen count [6]. To understand how the host immune system responds to tumor somatic mutations, we studied the association of infiltrating immune cells and total mutation load. Tumor purity is a confounding factor in this analysis, since purity affects the power to detect somatic mutations [20] and drives the pattern of gene expression [16]. After correcting for purity, we observed positive correlations between the total mutation count and infiltrating immune cells in a subset of cancers (Fig. 4a). In lung squamous cell carcinoma, lower grade glioma, HPV-negative head and neck cancer, and prostate cancer, CD8 T-cell infiltration increases significantly with tumor mutation load, in support of a previous study [6]. In addition, we observed that dendritic cell infiltration is correlated with the total mutation load in breast cancer (Spearman’s ρ = 0.11, q = 0.037), as is B-cell infiltration (ρ = 0.13, q = 0.018), suggesting cancer-specific roles for these cell types in antitumor immunity. Similar associations were observed when we used neoantigen load estimated from a previous work [6] instead of total mutation load (Additional file 1: Figure S5), corroborating our conclusion that increased tumor neoepitope load elevates the infiltration of multiple immune cell types.

Besides point mutations, microsatellite instability (MSI) is seen in colorectal, stomach, and endometrial cancers. MSI typically generates small indels across the genome, producing non-self antigens that may be recognized by the host immune system. Consistent with a previous report [7], we found CD8 T cells to be significantly more abundant in MSI-high (MSI-H) tumors compared with MSI-low (MSI-L) tumors in colon cancer (Fig. 4b). Among the remaining three TCGA cancers with available MSI information, we also found higher levels of MSI to be associated with increased CD8 T cells in stomach cancer. A recent study reported that MSI-high colon cancer patients showed significantly better responses to PD-1 blockade therapies [12] and our results suggest that this conclusion may be extended to other gastro-intestinal cancers with MSI.

To further investigate the regulation of immune infiltrates in different cancers, we also systematically studied the expression levels of chemokines and receptors. Most of these molecules were expressed in the microenvironment (Additional file 1: Figure S6a, b). CD8 T-cell level is significantly associated with a subset of chemokine–receptor pairs, including *CCL3*,*4*,*5*–*CCR1*,*5* and *XCL1*,*2*–*XCR1* (Fig. 4c). On the other hand, different molecules are associated with macrophage abundance in cancer-specific patterns. Macrophage infiltration appears to be related to *CXCL12*–*CXCR4* in thyroid, head and neck, stomach, and colon cancers and to *CCL14*,*CCL23*–*CCR1* in lung cancers (Fig. 4d). Our results highlight potential bases for inter-tumor heterogeneity in immune cell infiltration and suggest possible means for reducing macrophage recruitment to the tumor microenvironment.

### Implications for cancer immunotherapy targets

**Fig. 5:**
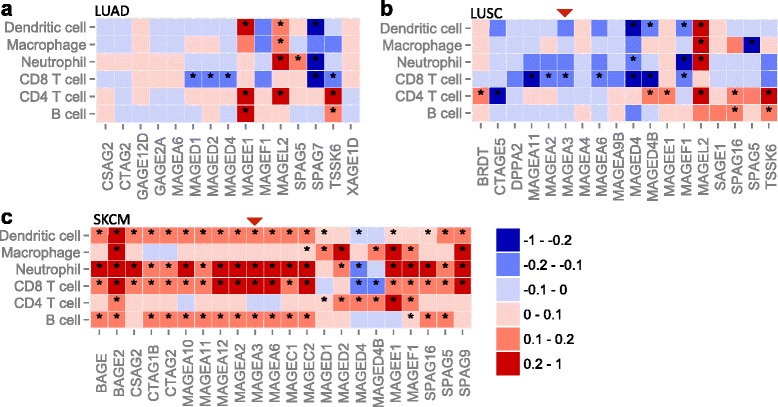
Association of immune cell infiltration and cancer/testis (CT) antigen expression in non-small cell lung carcinomas (**a**-**b**) and melanoma (**c**). Only genes with expression levels positively correlated with tumor purity (Pearson’s r > 0.1, q < 0.1) were selected to ensure cancer cell-specific expression. The heatmap presents correlations of gene expression and tumor infiltrating immune cells, which were calculated using partial Spearman’s correlation correcting for tumor purity. *Asterisks* indicate events significant at a 15 % FDR. *Red arrowheads* point to *MAGEA3*

We next focused on how antitumor immunity impacts cancer immunotherapies. First, we examined 109 known cancer/testis (CT) genes [6, 30] for their association with immune components. *MAGEA3* is an antigen that has been tested in cancer vaccines, although it failed to demonstrate improved progression-free survival in randomized non-small cell lung carcinoma clinical trials [31]. To explore the potential cause, we examined TCGA gene expression data in lung cancer. *MAGEA3* is not expressed in adenocarcinoma but is expressed in squamous cell carcinoma (Pearson’s correlation with purity >0.1, q < 0.1), although *MAGEA3* expression in the latter is negatively associated with CD8 T-cell infiltration (Fig. 5a, b). One possible interpretation of these results is that the host immune system in lung cancer patients fails to recognize *MAGEA3* as a neoantigen, which might explain the ineffectiveness of *MAGEA3*-based vaccines in lung cancer. In contrast, clinical trials of *MAGEA3* have been more successful in melanoma [31], consistent with our observed positive correlation between *MAGEA3* expression and CD8 T-cell abundance (Fig. 5c). Another positively correlated antigen in melanoma is the CT gene *NY-ESO-1* (*CTAG1B*), which was reported to be an effective vaccine antigen in a recent study [32]. We also studied prostate cancer, for which there is a US Food and Drug Administration-approved cell-based cancer vaccine, sipuleucel-T. As expected, many CT genes positively associate with CD8 T-cell level, implying strong host immune reactions to cancer antigens (Additional file 1: Figure S7). These observations suggest that association with infiltrating immune cells could be a useful criterion for selecting putative cancer vaccine targets, although the utility of this criterion awaits future experimental validation. Based on this hypothesis, we prioritized a list of CT genes in each cancer type through associations with infiltrating immune cells (Additional file 5: Table S4). This analysis suggested *SPAG5* as a potential candidate for future vaccine development in melanoma, glioma, prostate, bladder, breast, and head and neck cancers (Fig. 5c; Additional file 1: Figure S7). In TCGA, the median *SPAG5* expression in prostate, bladder, breast, and head and neck cancers (where normal tissues are available) is at least threefold over normal, suggesting that *SPAG5* is a potentially valid CT antigen in those cancer types. We have made these results available in TIMER and users can check the relationships between a given CT antigen and the levels of tumor infiltrating immune cells in different cancer types. In addition to known CT genes, we applied the same analysis procedures to a total of 2094 cancer-specific genes (“Methods”) for their expression associations with CD8 T-cell infiltration (Additional file 1: Figure S8; Additional file 6: Table S5). Ranked by the number of cancers for which this association is observed, the top 100 genes are most enriched in the gene ontology term “cell cycle” (false discovery rate (FDR) = 4.74 × 10^−24^), consistent with a previous report that expression of cell cycle genes upregulates immune infiltration [33].

The recent clinical success of checkpoint blockade drugs in treating metastatic melanoma [1] is an exciting development but predictive biomarkers are needed. In order to find promising targets in diverse cancer types, we examined how tumor-infiltrating immune cells correlate with inhibitory molecules, including the receptors *CTLA4*, *PD-1*, *LAG3*, and *TIM3*, and the ligands *PD-L1/2*, *B7-H3/4*. We noticed that the abundance of CD8 T cells correlates with the expression levels of inhibitory receptors in almost all cancers (Additional file 1: Figure S9a), indicating that inhibitory receptors are expressed in the infiltrating T cells of most tumor sites at the time of clinical intervention. We next investigated the potential cell sources of the inhibitory ligands. *PD-L1/2* and *B7-H3* expression positively correlates with macrophage infiltration in almost all cancers, suggesting TAM as a source of these ligands. The same is true for *B7-H4* except for gliomas (GBM and LGG), rectal cancer, and melanoma (Additional file 1: Figure S9b). In LGG and cervical cancer, further analysis reveals that *B7-H4* is expressed primarily in cancer cells (Additional file 1: Figure S9c). These findings might help identify alternative therapeutic options in different cancers.

**Fig. 6:**
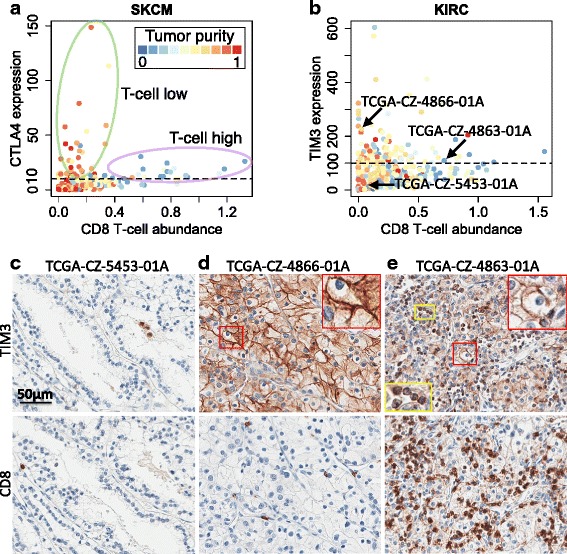
Varied levels of CD8 T-cell infiltration in tumors highly expressing inhibitory receptors. **a**, **b** High CTLA4/TIM3-expressing tumors in melanoma/KIRC show different CD8 T-cell infiltration levels. *Dashed lines* in both panels are the hypothetical high CTLA4 or TIM3 cutoff. Tumor purity is indicated by *color. Arrows* in **b** point to selected TCGA samples for immunohistochemistry (IHC) analysis. **c** Sample with low TIM3 expression and CD8 T-cell infiltration used as a negative control. TIM3- or CD8-expressing cells are brown in color. Selected samples with (1) high TIM3 expression and (2) low (**d**) or high (**e**) CD8 T-cell infiltration showed the existence of two KIRC sample groups. TIM3 expression in **d** is twice as high as in **e** according to RNA-seq data. **d** Image represents about 15 % TCGA KIRC samples while **e** represents 5 %. The *upper* and *lower panels* were synchronized. TIM3 was expressed in cancer cells (**d**, **e**) as well as in lymphocytes (**e**). High magnification insets are presented in **d** and **e** to illustrate TIM3 expression in different cell types. *Yellow boxes* indicate lymphocytes; *red boxes* indicate tumor cells

Although effective in a subset of patients, checkpoint blockade drugs usually have moderate response rates [31]. To explore possible explanations for the varied clinical responses, we studied the levels of inhibitory receptors and CD8 T-cell infiltration within each cancer type. Unexpectedly, we found that *CTLA4* expression in melanoma differentially correlates with CD8 T-cell levels in different tumors (Fig. 6a). In a subset of high purity tumors, *CTLA4* is highly expressed despite low levels of CD8 T cells. The same phenomenon holds for renal clear cell cancer, where tumors with high *TIM3* expression have varying CD8 T-cell levels (Fig. 6b). Since antibodies that allow immunohistochemical staining of *CTLA4* were unavailable, we sought to experimentally validate the levels of *TIM3* and CD8 T-cell infiltration. A subset of the TCGA renal tumors was submitted locally and remaining tissue slides were available for these. Staining of selected renal tumors (Fig. 6c–e) confirmed that *TIM3* is expressed on cancer cells as well as infiltrating lymphocytes (Fig. 6e), an observation recently reported [34]. More importantly, we found that tumors with high *TIM3* expression can be divided into two distinct groups with different levels of infiltrating CD8 T cells (Fig. 6d, e; Additional file 1: Figure S10). Melanoma and kidney cancer with high expression of inhibitory receptors and low levels of CD8 T-cell infiltration may have different clinical responses to checkpoint blockade drugs compared with tumors with high CD8 T-cell infiltration, a hypothesis that awaits further testing.

## Discussion

With the clinical success of cancer immunotherapies, there is a growing need for a comprehensive understanding of tumor–immune interactions. In this study, we developed a novel method for tumor immune cell deconvolution and have provided a comprehensive catalog of the abundance of six immune infiltrates in 23 cancer types. Our method was validated using Monte Carlo simulations, orthogonal estimates from DNA methylation-based inferences, as well as pathological assessment. Further validations using immunohistochemistry (IHC) or cell sorting are infeasible since TCGA does not provide original tumor samples. We have made our estimated immune cell abundance together with associated findings available as a public resource, TIMER, for biomedical researchers to address more interesting questions in cancer immunology. The information covered in this work was accessible through a user-interactive website (http://cistrome.org/TIMER).

Our work first provided a systematic prognostic landscape of different tumor-infiltrating immune cells in diverse cancer types. We compared our results with two recent studies on the same topic [6, 11]. The method used in Gentles et al., CIBERSORT [35], is currently only applicable to microarray data, thus unable to analyze the TCGA RNA-seq data. Therefore, our immune component estimation is a unique addition to TCGA for future integrative analyses of tumor–immune interactions. By including more immune cell types into regression, CIBERSORT inference also suffered from statistical co-linearity that might have resulted in biased estimations (Additional file 7: Table S6; Additional file 8). Due to this limitation, although Gentles et al. studied more cell types, they reported few significant prognostic immune predictors, without correction for other clinical confounders. In contrast, we observed many more significant clinical associations with the correction of multiple cofactors. It should be noted that due to limited sample size, some of these associations only reached a FDR of 0.15, yet 85 % of these significant calls are expected to be true and still be informative. These observations include both established results from previous clinical studies as well as novel ones that may provide new angles to study the clinical responses of immunotherapies.

We then demonstrated the usefulness of TIMER by studying putative immunotherapy targets and made several interesting observations. First, CD8 T-cell and macrophage infiltration is likely to be regulated by different sets of chemokine and chemokine receptors in different cancers. Second, the effectiveness of cancer vaccine targets might be predicted via association with immune infiltration levels; based on our data, it appears that *SPAG5* is a potential vaccine candidate for multiple cancers. Third, the correlation of *CTLA4* and *PD-1* expression with CD8 T-cell abundance suggests that a subset of patients from most cancer types may benefit from combined use of anti-CTLA4 and anti-PD-1 agents. Finally, *CTLA4* and *TIM3* expression fall in distinct groups relative to CD8 T-cell infiltration in melanoma and kidney cancer, respectively, which might contribute to the varied clinical response to checkpoint blockade therapies. Although detailed characterization of the underlying mechanisms requires further work, the findings from this study have immediate implications for cancer immunotherapies.

The current release of TIMER is based on estimations using transcriptome profiles (RNA-seq or microarray) from whole tissues at a single time point. Consequently, TIMER might have limited relevance to distinguish stromal or intra-tumor immune cell localization or capture tumor cell heterogeneity. In the future, we anticipate more experimental measures with improved spatial and temporal resolutions, and the applicability of TIMER should continue to grow as we make inferences on new datasets and incorporate them into the existing resources.

## Conclusions

In this study we systematically documented the abundance of six tumor-infiltrating immune compartments for TCGA samples and integratively analyzed the immune infiltration with other cancer molecular profiles. We identified widespread clinical associations of different immune cell types in multiple cancers. Systematic exploration of tumor–immune interactions revealed cancer genetic alterations and chemokine/receptor expression networks are potential regulators of immune cell infiltration heterogeneity. Our analyses on putative immunotherapy targets led to the findings on cancer vaccine candidate *SPAG5* and dichotomized CD8 T-cell levels in tumors highly expressing inhibitory receptors. Our results add value to the current knowledgebase of tumor immunity and provide a public resource for further exploration of cancer–immune interactions.

## Methods

### Data collection and preprocessing

Molecular data for 23 TCGA cancer types, including level 2 DNA SNP array and clinical data, were downloaded from TCGA data portal (https://gdc.nci.nih.gov) and level 3 mRNA expression data from the GDAC Firehose website (http://gdac.broadinstitute.org). For all cancers but GBM or OV, whole transcriptome RNA-sequencing (RNA-seq) data were available and we used the RSEM-processed transcript per million (TPM) measure. For GBM and OV, where RNA-seq data were available for only a subset (approximately one-third for GBM and one-half for OV) of samples, we used microarray data profiled using Affymetrix HGU133a platforms for immune component estimation (Additional file 8). In this study, we found that the HGU133a array could not accurately profile the lowly expressed genes (including important therapeutic targets such as PD-1). Therefore, we applied RNA-seq data for GBM and OV to study the immunotherapy targets (Fig. 5; Additional file 1: Figures S7 and S9). We used the Human Primary Cell Atlas (HPCA) [36] as the reference dataset of gene expression profiles of sorted immune cell types. HPCA is a collection of previous analyses on human primary cells using the Affymetrix HGU133plus2 platform and includes more than 100 studies, which are numbered in the dataset. We selected six immune cell types for our downstream analysis and the studies used for each cell type are: 25, 45, and 115 for B cells; 12, 42, 76, and 115 for CD4 T cells; 42, 115, and 116 for CD8 T cells; 39, 62, and 77 for neutrophils; 104 for macrophages; and 7, 9, 14, 28, 86, 89, 91, and 103 for dendritic cells. A complete list of reference samples is available in Additional file 9: Table S7. It should be noted that each immune cell type still represents a mixed population with cells of potentially distinct functions. For example, CD4 T cells may include helper T cells, memory T cells, and regulatory T cells and B cells may represent a mixture of mature CD19 B cells and B plasma cells. In this study, we do not seek to further distinguish these subpopulations, as their expression profiles are highly similar. Signature genes (n = 2271, denoted as *G*_i_) overexpressed in the immune lineage were obtained from the Immune Response *In Silico* database [18].

### Inclusion criteria for immune cell types

In order to minimize co-linearity in the regression analysis and maximize the robustness of our inference, our study focused on six immune components based on two criteria. First, the reference data contain at least ten independent samples of the immune cell type. Second, if the expression profile of a given cell type is highly correlated (sample-wise Pearson’s r ≥ 0.9) with other cell type(s), we chose the cell type with more samples. The selected cell types represent the finest resolution of immune cell lineages that we can achieve based on the above inclusion standards. Cell types excluded from the inference may affect the highly correlated immune components included. Improved reference immune datasets will be needed to deconvolve individual cell types.

### Computational method for immune cell composition deconvolution

We first estimated sample purity for each tumor through DNA SNP array data using our previously developed tool *CHAT* [15]. Genomic estimations of tumor purity have been validated using diluted series of cancer and blood cell lines with known mixture ratios [16]. To clarify, the quantity inferred from *CHAT* is the fraction of aneuploid cells. In this work, we used this quantity as a surrogate of tumor purity. Samples with percent on point (PoP) <0.01 were excluded, where PoP is a quality measure reported by *CHAT*. In each cancer, we selected genes with expression values negatively correlated with tumor purity (Pearson’s r ≤ −0.2, *P* ≤ 0.05), denoted as *G*_p_, and intersected with *G*_i_. Our goal in this step is to select informative genes with expression levels strongly affected by tumor purity. It is not important whether the gene is highly or lowly expressed. Pearson’s correlation is suitable because it is a measure of the linear dependence of two random variables, disregarding the magnitude of the observations. The resulting smaller gene set intersection was denoted as *G*_0_, which is cancer-type specific. Meanwhile, we merged the tumor gene expression with the reference immune cell data of all genes using ComBat [17]. According to the principal components analysis plot, ComBat effectively removed the batch effect between different datasets generated using different platforms (Fig. 1b). For each of the six cell types, we then calculated the median expression value in all the samples available for that cell type for each gene in *G*_0_. The resulting dataset (referred to as ***R***) contains six vectors of gene expression values, each for one cell type. We used ***R*** to filter *G*_*0*_ in the following way: for each vector in ***R***, genes with expression values in the top 1 % in *G*_*0*_ are removed. This is because genes expressed at extremely high levels in the reference dataset will dominate the inference results. Since highly expressed genes have large variance, our inferences become very sensitive to these outliers. We therefore remove them to acquire more robust estimations. The total removed genes were the union of the top 1 % from of the six vectors. The resulting gene set was denoted as *G*_*0f*_. For each sample, a constrained least square fitting described in a previous study [19] was applied to infer the relative abundance for each of the six immune components (Fig. 1e). The predictions of this method were validated using mixtures of different blood cell types with known ratios. For a given sample, let Y^g^ denote the gene expression of gene *g*, where *g* ∈ *G*_*0f*_. Let X^g^_r_ denote the gene expression of gene *g* in immune cell type r (r = 1,2,…,6) in reference dataset ***R***. Since we model that the given sample is a mixture of the six immune cell types, our task is to find positive coefficients ***f*** to minimize the total squared differences:

$$ f=\underset{\forall r:{f}_r>0}{\mathrm{argmin}}\underset{g\in \left\{{G}_{0f}\right\}}{\varSigma }{\left({Y}^g-{\displaystyle \sum_{r=1}^6{f}_r{X}_r^g}\right)}^2 $$

which is a constrained linear regression problem. The estimation accuracy of ***f*** should be affected by the genes used in the fitting, although our model assumes that such an ***f*** exists and should be the same for all genes. To note, coefficients ***f*** estimated using this method are the relative abundance of immune cells. The scale of the estimation of an individual immune cell type is determined by the variance of the corresponding reference data X_r_. Therefore, ***f*** are not comparable between cancer types or different immune cells. Source codes for TIMER and downstream statistical analysis as well as related data files are available at http://cistrome.org/TIMER/download.html.

### Correction for reference immune cell colinearity

Although the six immune cell types in this study are selected in such ways that the colinearity between cell types is minimized, we found that in THCA and UCS, CD4 and CD8 T-cell signatures are still very similar. Consequently, the inferred CD4 and CD8 T-cell levels are negatively correlated (Pearson’s r ≤ −0.3), which is an artifact of covariates’ colinearity in the constraint regression. Additional analysis on these two cancers revealed that the negative correlation is driven by a small number of CD4 or CD8 T-cell signature genes that are extremely overexpressed in the tumor samples. We remove the union of the top expressed gene in each tumor sample and re-estimate ***f***. This step is repeated until the correlation between estimated CD4 and CD8 T-cell levels is larger than −0.3. This analysis provides more robust estimations of immune cell abundance in cancer types.

### Pathological estimation of neutrophil infiltration in BLCA

For the TCGA data sets, the original samples are unavailable for further studies; however, hematoxylin and eosin (H&E) digital slides have been publicly released. While it is not possible to distinguish T cells and B cells by H&E, neutrophils are morphologically distinctive and their abundance can be estimated. Occasional dendritic cells and macrophages can be identified by H&E but their true abundance is difficult to estimate in the absence of immunohistochemical stains. Slides were reviewed via digital images viewed with the Cancer Digital Slide Archive (http://cancer.digitalslidearchive.net). We chose BLCA because it has a large sample size (n = 404), does not have excessive necrosis, and has sufficient neutrophil counts and sample variety to allow for validation by histological evaluation. The pathologist reviewing the slides was blinded to the in silico neutrophil predictions. Samples were stratified into three groups (high, medium, low) with levels of neutrophils relative to the entire collection of samples.

### DNA methylation-based total leukocyte estimation

The percentage of total DNA for each tumor was estimated using DNA methylation data as previously described and compared with purity estimates derived from SNP data [20, 37]. In brief, the estimates were derived from loci with tissue-specific methylation that distinguishes the corresponding tissue type from lymphocytes using the TCGA Pancan12 [38] DNA methylation dataset. The leukocyte methylation signature was derived as follows. Each probe was ranked by the difference in mean beta value in buffy coat and corresponding normal samples. We retained the 100 probes with the largest positive difference and the 100 with the largest negative difference between mean DNA methylation in normal tissues and peripheral blood leukocytes, designated *NT* and *BC* probes (hypermethylated in normal tissue compared with buffy coat, and vice versa, respectively). Let T_ik_ denote the beta value for probe *k* in tumor sample *i*. Let B_k_ denote the average beta value of buffy coat samples for each probe. Let T_k_ denote the minimum observed beta value across all tumor samples for the *BC* probes and the maximum for the *NT* probes, which theoretically reflects the ground state of methylation level in the purest tumor. Denote with f_B_ the fraction of buffy coat (leukocyte) components in the sample, then, assuming a linear relationship, we have the following equation for each probe:

$$ {\mathrm{T}}_{\mathrm{ik}} = {\mathrm{B}}_{\mathrm{k}}{\mathrm{f}}_{\mathrm{B}} + {\mathrm{T}}_{\mathrm{k}}\left(1 - {\mathrm{f}}_{\mathrm{B}}\right) $$

Solving this equation for f_B_ gives:

$$ {\mathrm{f}}_{\mathrm{B}} = \left({\mathrm{T}}_{\mathrm{ik}} - {\mathrm{T}}_{\mathrm{k}}\right)/\left({\mathrm{B}}_{\mathrm{k}} - {\mathrm{T}}_{\mathrm{k}}\right) $$

The values of f_B_ for each of the 200 probes in the signature were calculated and a kernel density estimate of f_B_ was obtained. The leukocyte signature was then calculated as the mode of this density estimate.

### Monte Carlo sampling and in silico validation

We validated our predictions on infiltrating immune cell abundance using in silico simulated data. As mentioned, for each cancer we selected a gene set *G*_*0f*_ (length n_0_) for least squares fitting. In order to control for the mixing ratios of the six components while maintaining the correlation structure of the real data, we first calculate the gene–gene covariance matrix ***Σ***_*0f*_ for all the genes in *G*_*0f*_ using tumor expression data. We then randomly sample six numbers *f*_*1-6*_, from Uniform(0,1). We calculate ***μ***_*0f*_ (length n_0_), which is the average of six immune components weighted by *f*_1-6_. Next, we sample a vector of length *n*_*0*_ from multivariate normal distribution with mean ***μ***_*0f*_ and covariance ***Σ***_*0f*_. For each cancer type, we simulated the same number of samples as its sample size in the TCGA data. After applying our method, we compared the estimated immune abundance with true values using Pearson’s correlation. Low quality estimations with Pearson’s r ≤ 0.2 were excluded from the downstream analysis.

### Selection of cancer specific genes

For each cancer type, we compare tumor samples with all normal samples collectively. Only genes overexpressed in tumor samples and absent or expressed at lower levels in all normal tissues were selected. Differentially expressed genes were selected based on a FDR ≤0.05 and at least a twofold difference in expression levels. In the case of tumors with established clinical subtypes, such as breast cancer, we selected the top 25 samples for each gene based on their rank of raw read counts, then identified differentially expressed genes within each subtype. The final tumor-specific gene set was the union of all the cancer types (or subtypes).

### Statistical analysis

Multivariate Cox regression, log-rank test and Kaplan–Meier estimators were implemented using the R package *survival*. The association between CD8 T-cell abundance and tumor status was evaluated using logistic regression corrected for age and clinical stage and was implemented using the R package *glm*. The same analysis was performed for neutrophil abundance and gender associations, corrected for age and smoking history. Partial correlations of immune cell abundance and gene expression of chemokines and receptors, somatic mutation counts, CT gene expression, as well as immunosuppressive molecule expression were calculated using the R package *ppcor*. Multiple test correction was performed using the R package *qvalue* [39] and FDR thresholds are applied based on the abundance of signals in the data. In this study, we applied the Pearson correlation to purity and gene expression because it is reasonable to expect that the expression level is linearly associated with tumor purity. For others, we used the Spearman correlation. We applied partial correlation analysis to remove the influence of tumor purity on the involved variables. All other analyses, including linear regression, Fisher’s exact test, Wilcoxon rank sum test, Spearman’s correlation, and hierarchical clustering, were performed using R [40]. Of note, in Figs. 2b and 3b, we used the 20 percentile as a cutoff only to help visualize the association of immune infiltration with outcomes and the statistical significance was determined by multivariate Cox regression (Fig. 3a) including all the samples. Our results on survival analysis, neoantigen association, tumor recurrence, and association of checkpoint blockade inhibitory molecules with immune cells are available in Additional file 10: Table S8.

### Additional analysis on HNSC and SKCM

One intriguing result we observed is that univariate and multivariate survival analysis results for HNSC and SKCM are not consistent (Fig. 3a, b). For HNSC, we discovered that HPV infection, a recently identified prognostic factor [41], correlates with CD8 T-cell infiltration (Additional file 1: Figure S4). It is likely that the previously observed association of CD8 T cells with survival is contributed to by virus infection. On the other hand, for SKCM, we found that the infiltration level of CD8 T cells is highly correlated with neutrophils (Pearson’s r = 0.79) and dendritic cells (r = 0.81), indicating that these immune cells work in concert. As highly correlated features confound each other in a multiple regression, we performed principal component analysis on the abundance of the six immune cells. We reanalyzed the Cox model using six principal components (PCs), age and stage as covariates, and found PC1 (hazard ratio (HR) = 2.6 × 10^−4^, *p* = 0.0062), PC4 (HR = 1.07 × 10^2^, *p* = 0.033) and PC6 (HR = 0.01, *p* = 0.008) to be significantly associated with survival. PC1 was comprised of CD8 T cells, neutrophils, and dendritic cells (by Pearson’s correlation), thus capturing the colinearity in the data. PC4 represented macrophages and predicted worse outcome. There was no clear assignment of PC6 to any immune component(s) and it may represent an unselected immune cell type.

### Additional analysis on OV and BRCA

In the survival analysis (Fig. 3a, b), we failed to identify some known prognostic predictors, notably B cells in OV and CD8 T cells in BRCA. A previous study reported that CD20 cells positively associate with survival [42]. We investigated the expression levels of the B-cell markers CD19 and CD20 in OV and discovered that tumor purity is not negatively correlated with gene expression levels for both genes, indicating that aneuploid cells in ovarian cancer may also express B-cell markers. Therefore, cell sorting based on CD19 or CD20, which is not the B-cell component in our analysis, is likely to select cancer cells. CD8 T cells were previously reported to associate with better outcome in BRCA [43], although we did not observe this relationship. This is possibly due to insufficient follow-up time or fewer deaths in the TCGA BRCA data, which thus underpowered our survival analysis.

### Patient samples for IHC

De-identified clear cell renal cell carcinoma (ccRCC) formalin-fixed and paraffin-embedded tissue blocks from cases included in the TCGA KIRC cohort were obtained from the department of Pathology at the Brigham and Women’s Hospital. Patients had provided an informed consent for use of specimens and baseline and prospective clinical data for research purposes. The study was approved by the Dana-Farber/Harvard Cancer Center (DF/HCC) institutional review board. In total, five TCGA samples were selected for IHC: TCGA-CZ-5453-01A (negative control), TCGA-CZ-4866-01A, TCGA-CZ-4863-01A, TCGA-CZ-5459-01A, and TCGA-CZ-4862-01A.

### IHC protocol

IHC for *TIM3* and CD8 expression was performed as described below. For *TIM3* IHC, rehydrated tissue sections were boiled in EDTA buffer (pH 8) with a microwave at 92 °C for 30 minutes. After cooling down at room temperature (RT), tissue sections were successively incubated with a peroxidase block (Dual Endogenous Enzyme Block, Dako) and a protein block (Serum Free Block, Dako) for 10 minutes each. Sections were next incubated for 1 h at RT with the goat polyclonal anti-TIM3 antibody (1/400, AF2365, R&D Systems) diluted in Da Vinci Green Diluent (Biocare Medical). Tissue sections were then incubated with a rabbit anti-goat biotin-conjugated antibody (1/750, Dako) for 30 minutes followed by an incubation of 30 minutes with EnVision anti-rabbit horseradish peroxidase (HRP)-conjugated antibody (Dako). The HRP visualization was performed by applying 3,3-diaminobenzidine substrate (Dako) for 5 minutes. Nuclei were counterstained with hematoxylin. For CD8 staining, rehydrated tissue sections were boiled in EDTA buffer (pH 8) with a pressure cooker at 125 °C for 30 s. Sections were blocked as described above and then incubated for 1 h at RT with a mouse monoclonal anti-CD8 antibody (1/100, clone C8/144B, Dako) diluted in Antibody Diluent with Background Reducing Components (Dako). Sections were then incubated with EnVision anti-mouse HRP-conjugated antibody for 30 minutes (Dako). The HRP visualization and the counterstaining were performed as described above.

## Abbreviations

ACC, adenocortical carcinoma; BLCA, bladder carcinoma; BRCA, breast carcinoma; CESC, cervical squamous carcinoma; COAD, colon adenocarcinoma; CT, cancer/testis; CTL, cytotoxic T cell; CYT, cytolytic activity; DLBC, diffusive large B-cell lymphoma; GBM, glioblastoma multiforme; H&E, hematoxylin and eosin; HNSC, head and neck carcinoma; HPCA, Human Primary Cell Atlas; HPV, human papilloma virus; HR, hazard ratio; HRP, horseradish peroxidase; IHC, immunohistochemistry; KICH, kidney chromophobe; KIRC, kidney renal clear cell carcinoma; KIRP, kidney renal papillary cell carcinoma; LGG, lower grade glioma; LIHC, liver hepatocellular carcinoma; LUAD, lung adenocarcinoma; LUSC, lung squamous carcinoma; MSI, microsatellite instability; OV, ovarian serous cystadenocarcinoma; PC, principal component; PRAD, prostate adenocarcinoma; READ, rectum adenocarcinoma; RT, room temperature; SKCM, skin cutaneous melanoma; STAD, stomach adenocarcinoma; TAM, tumor-associated macrophage; TCGA, The Cancer Genome Atlas; THCA, thyroid carcinoma; UCEC, uterine corpus endometrial carcinoma; UCS, uterine carsinosarcoma

## Additional files

Additional file 1: Supplementary figures. (PDF 9244 kb) Additional file 2: Table S1. Purity correlated genes. (XLSX 442 kb) Additional file 3: Table S2. Immune infiltration estimation. (XLSX 2364 kb) Additional file 4: Table S3. Clinical reports on CD8 T cells. (XLSX 43 kb) Additional file 5: Table S4. CT genes. (XLSX 60 kb) Additional file 6: Table S5. Additional cancer-specific genes. (XLSX 990 kb) Additional file 7: Table S6. Comparison with CIBERSORT. (XLSX 55 kb) Additional file 8: Supplementary information. (DOCX 84 kb) Additional file 9: Table S7. All statistical analysis. (XLSX 49 kb) Additional file 10: Table S8. HPCA cell index. (XLSX 43 kb)
