# Supplementary figures and images for: Comprehensive analyses of tumor immunity: implications for cancer immunotherapy
Source: Genome Biol. 2016 Aug 22;17:174. doi: 10.1186/s13059-016-1028-7 (PMC4993001; doi:10.1186/s13059-016-1028-7)

Figure S1

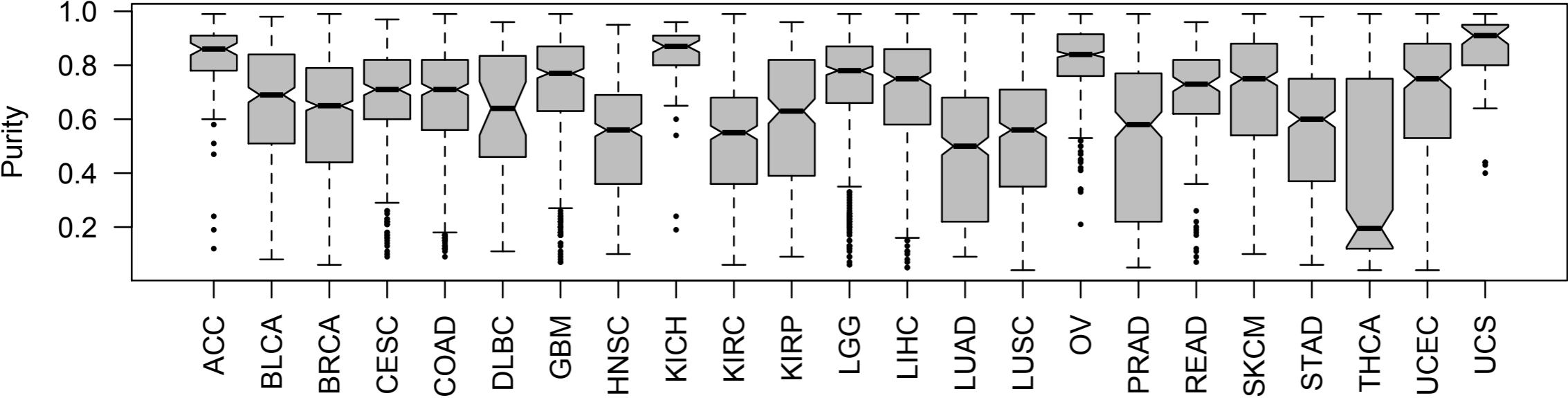

Figure S2

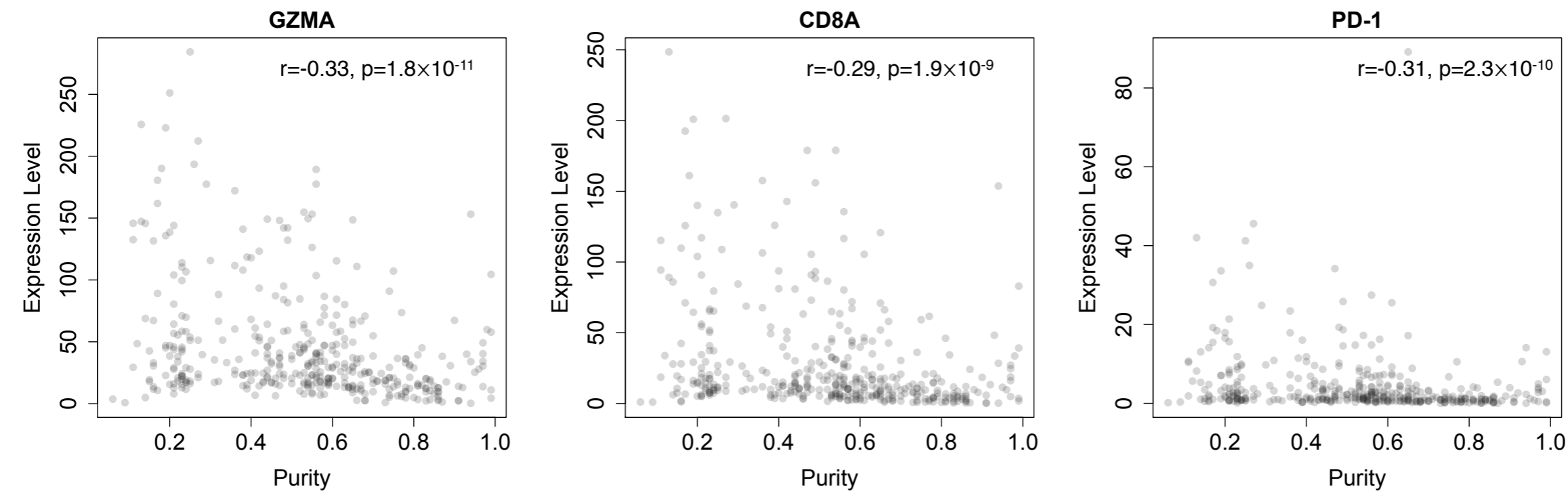

Figure S3

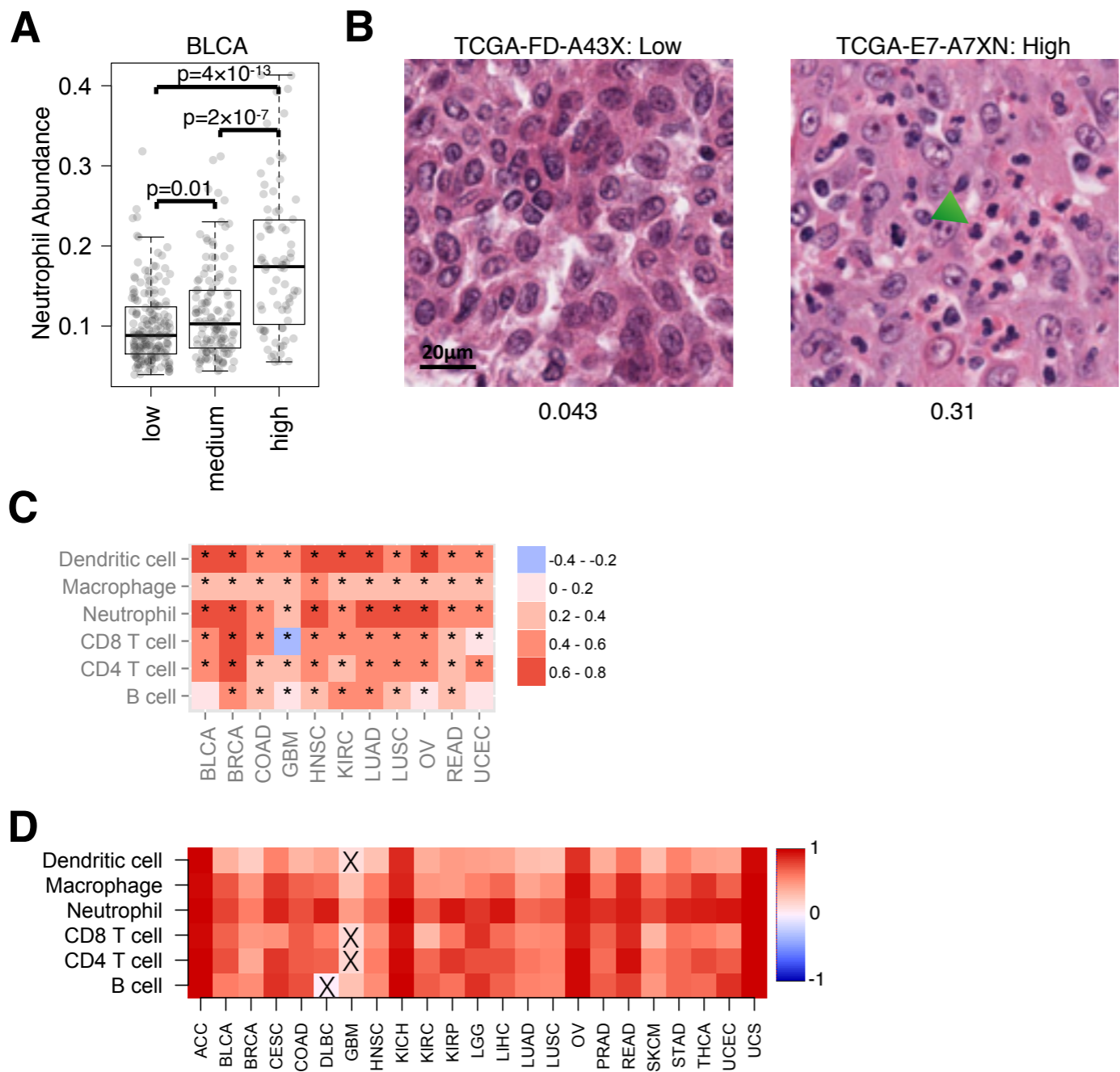

Figure S4

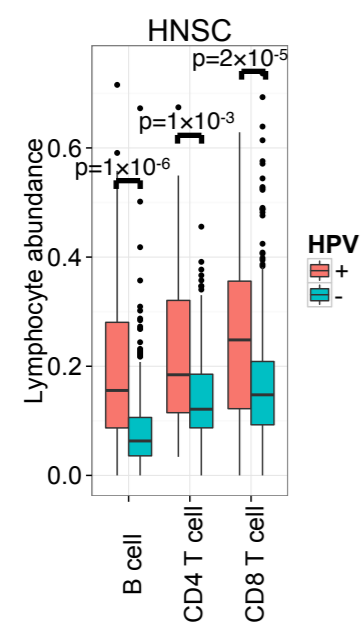

Figure S5

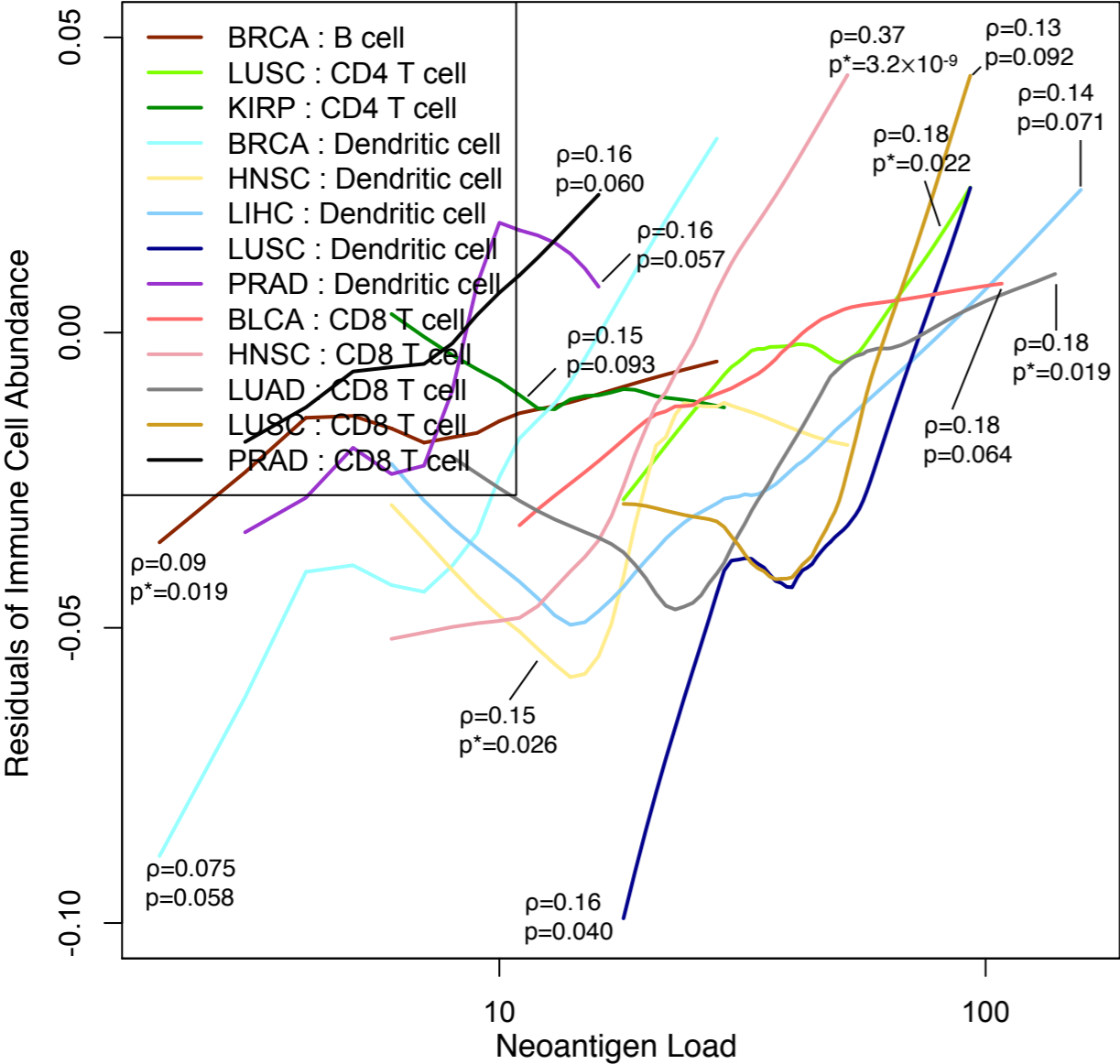

Figure S6

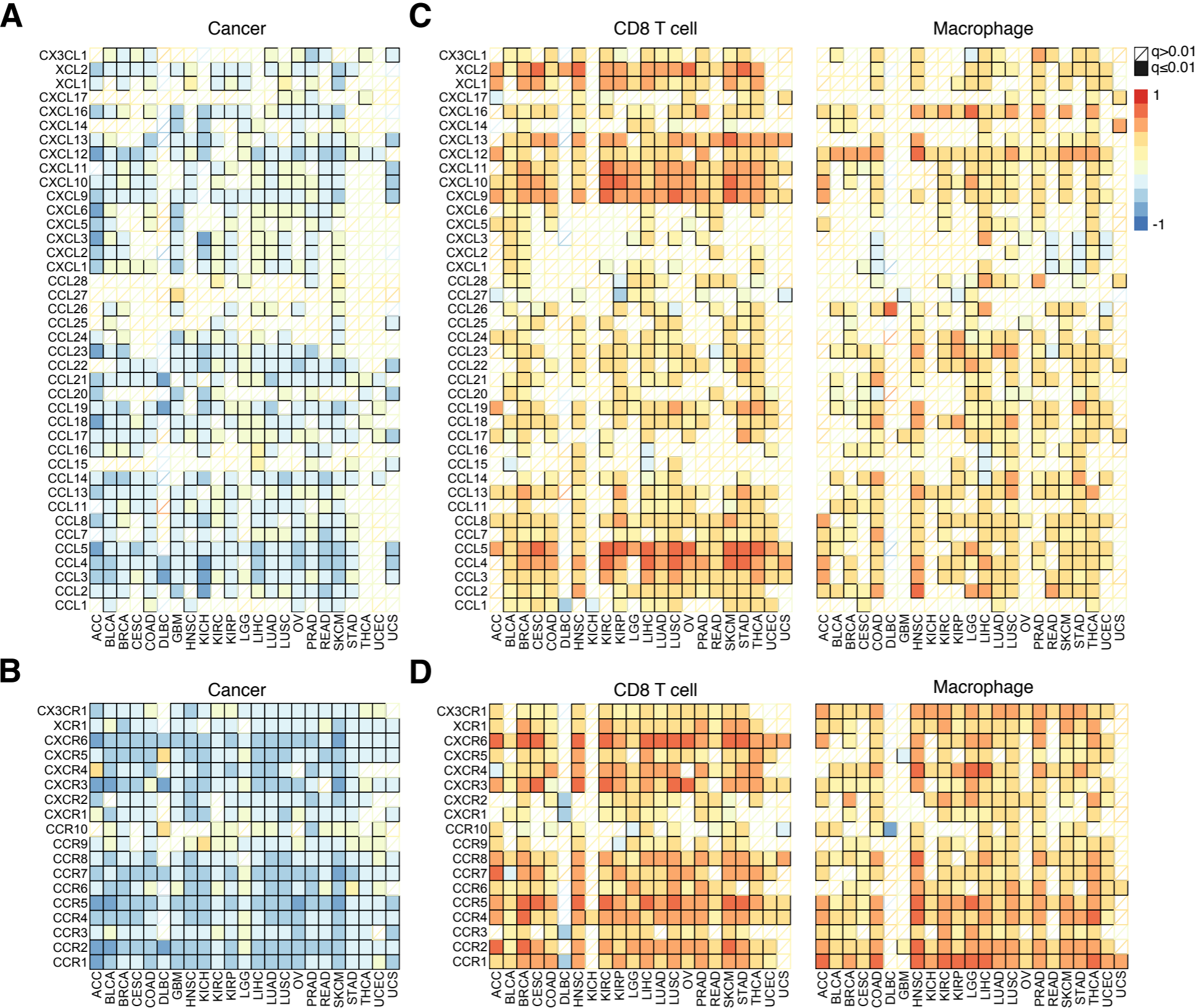

### Figure S7

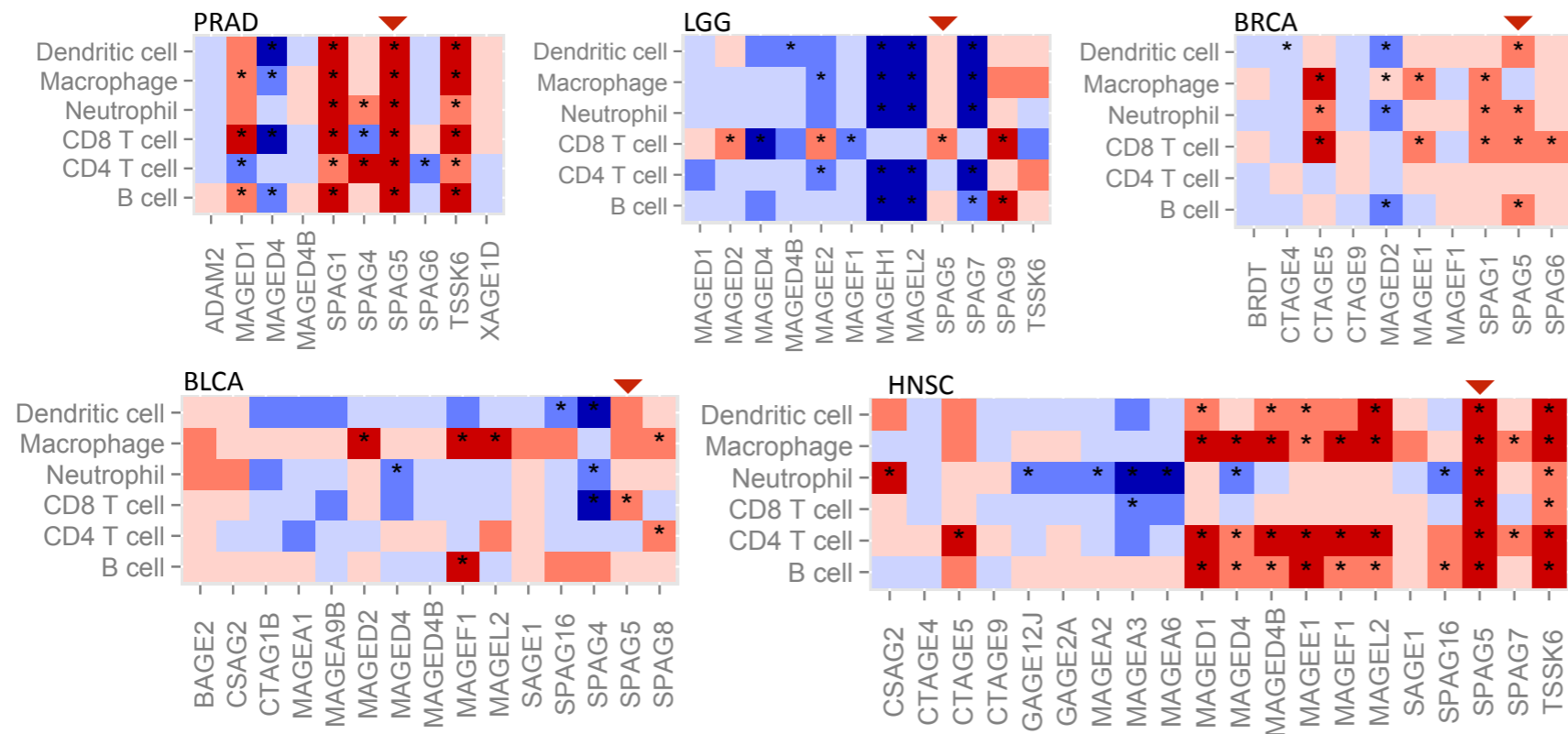

Figure S8

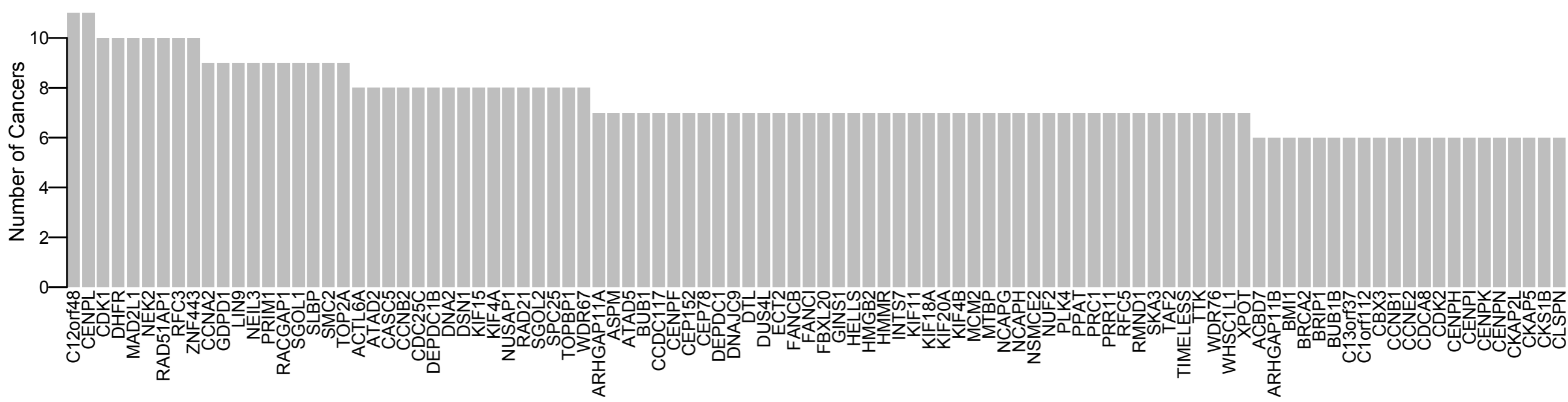

### Figure S9

**A**

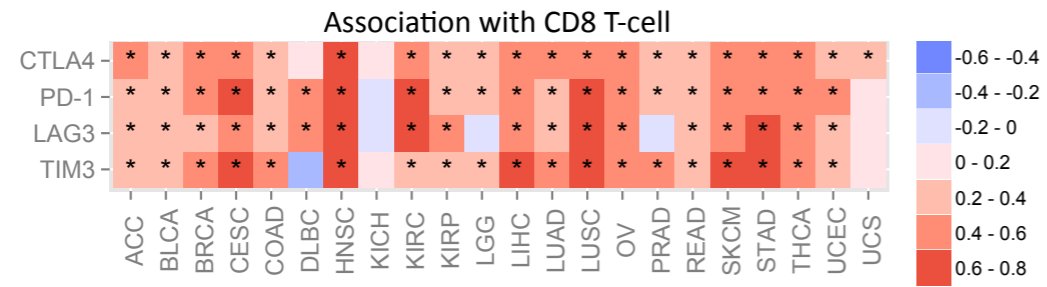

# B

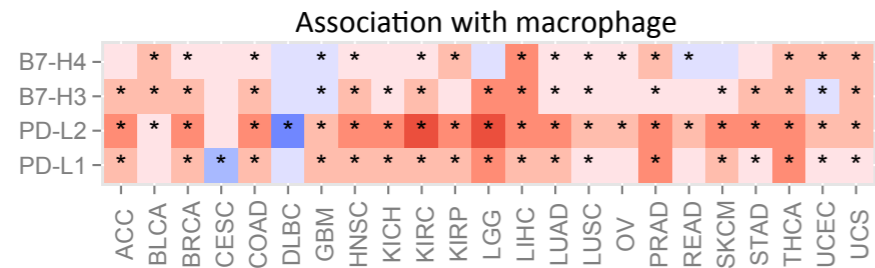

**C**

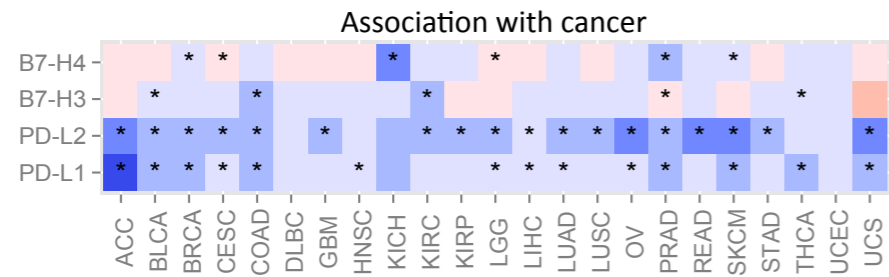

Figure S10

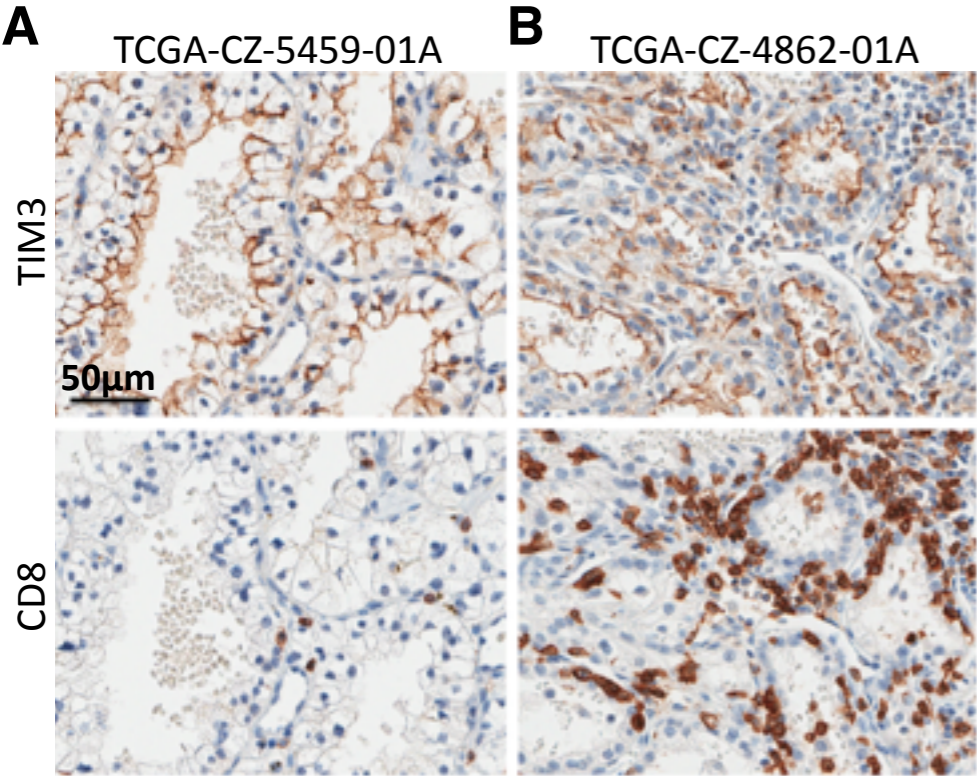

Supplement: Additional file 1: — Supplementary figures. (PDF 9244 kb) [file 13059_2016_1028_MOESM1_ESM.pdf]
