# Supplementary material for: Comprehensive analyses of tumor immunity: implications for cancer immunotherapy
Source: Genome Biol. 2016 Aug 22;17:174. doi: 10.1186/s13059-016-1028-7 (PMC4993001; doi:10.1186/s13059-016-1028-7)
Supplement: Additional file 8: — Supplementary information. (DOCX 84 kb) [file 13059_2016_1028_MOESM8_ESM.docx]

**Supplementary Information for Comprehensive analyses of tumor immunity: implications for cancer immunotherapy**

Bo Li^1,2^, Eric Severson^1,3^, Jean-Christophe Pignon^3^, Haoquan Zhao^1^, Taiwen Li^4^, Jesse Novak^3^, Peng Jiang^1^, Hui Shen^5^, Jon C. Aster^3^, Scott Rodig^3^, Sabina Signoretti^3^, Jun S. Liu*^,2^ and X. Shirley Liu*^,1^

^1^: Department of Biostatistics and Computational Biology, Dana Farber Cancer Institute, 450 Brookline Ave., Boston, MA, 02215

^2^: Department of Statistics, Harvard University, 1 Oxford St, Cambridge, MA 02138

^3^: Department of Pathology, Brigham and Women's Hospital and Harvard Medical School, 75 Francis St., Boston, MA, 02215

^4^: State Key Laboratory of Oral Diseases, West China Hospital of Stomatology, Sichuan University, 14 Renmin South Rd 3rd Section, Wuhou, Chengdu, Sichuan, China, 610041

^5^: Center for Epigenetics, Van Andel Research Institute, 333 Bostwick Ave N.E., Grand Rapids, MI 49503

*: Corresponding Authors

X. Shirley Liu, PhD

Phone: 6176322472

Email: xsliu@jimmy.harvard.edu

Jun S. Liu, PhD

Phone: 6174951600

Email: jliu@stat.harvard.edu

**Supplementary Discussion**

*B cell infiltration predicts better outcome in selected cancer types*

In the main text (Figure 2B), we found that in a subset of samples, B cell infiltration is enriched in the tumors compared with adjacent or normal tissues. Survival analysis suggests B cell infiltration predicts better outcome in 3 out of 8 of these cancer types, while not in the other five. The functional impact of B cell infiltration in solid cancers remains largely uncharacterized. Previous studies in animal models show contradictory results with those in humans [[1](#_ENREF_1)], and our study provides the first unbiased evaluation of this phenomenon in large patient tumor cohorts. Our results may reflect the complex nature of the tumor-infiltrating B cells in different cancer or tissue types.

Another possible reason to this heterogeneity is potentially that the B cell component in our analysis represents a mixture of population, including inactivated/activated B cells and plasma cells. In different cancer types, the composition of these B cell subtypes may be substantially different and result in different clinical responses.

*Cancer/testis gene negatively correlates with selected immune cell types*

In the main text (Figure 5), we found that some cancer/testis (CT) genes show negative correlation with immune cell infiltration levels after correcting for tumor purity. CT antigen expression is an indication of genome instability of cancer cells. One of the possible explanations of the negative correlation between *MAGEA3* expression and CD8 T cell infiltration is that tumors with higher genome instability develop advanced immune-escape mechanisms that void the infiltrating immune cells. A previous study observed significantly decreased activated CD8 T cell infiltration in higher stage colon cancer compared with lower stage [[2](#_ENREF_2)], which is in line with this explanation.

*Usage of Affymetrix hgu133a array in GBM and OV*

Gene expression of TCGA GBM and OV samples were profiled using Affymetrix HGU133a platform. Meanwhile, 160 out of 540 GBM and 296 out of 574 OV samples also have RNA-seq data available. We estimated the immune cell fractions using both RNA-seq and HGU133a array data in available samples in GBM and OV, and observed high concordance between the two estimations (data not shown), despite the artifacts of HGU133a platform. This is potentially due to the fact that our method utilized a large number of genes to make the inference and was insensitive to the lowly expressed genes inaccurately profiled by HGU133a array. In order to achieve the maximum power of survival analysis, we used HGU133a array to provide immune cell abundance estimations, as it has larger sample size. When comparing immune infiltration with the expression levels of individual genes, we used the more accurate RNA-seq data for these genes.

*Statistical Co-linearity of CIBERSORT Inferences*

CIBERSORT developed in a previous study deconvolves 22 immune cell types using a pre-defined gene signature matrix [[3](#_ENREF_3)] and applies to array data. In our study, we used CIBERSORT to estimate the immune infiltration levels in colon cancer, lung cancer, GBM and ovarian cancer. As the inference from CIBERSORT are reported to be additive, we aggregate the corresponding cell types into our 6 categories to directly compare with our results.

We first studied the correlations between the abundance of different immune components in colorectal cancer (CRC). It has been reported that different immune cells positively correlate with each other in the CRC microenvironment [[4](#_ENREF_4)]. This observation intuitively makes sense because immune cells work in synergy to eliminate foreign antigens and many chemokines involved in cell migration are shared by different immune cell types. We examined the correlations between cell types in our inference and discovered consistent positive correlations. In contrast, when examining the estimation by CIBERSORT, we found that most of the inter cell type associations are negative, an observation contradicts previous study (Table S6). These negative correlations between similar cell types (especially CD4 vs CD8 T cells and DC vs Macrophage) are also observed in lung, brain and ovarian cancer datasets by CIBERSORT but not by our estimations (Table S6).

In fact, during our initial method development, we observed very similar negative correlations between coefficients when we included more cell types. It is known that for regression based methods including ours and CIBERSORT, co-linearity resulted from similar covariates would generate coefficients that are negatively correlated. This correlation is a technical artifact, which should be carefully removed. Immune cells that closely related usually share very similar expression profiles, such as Th1, Th2, Th17 and Treg, which inevitably cause co-linearity when they are all included into the regression. Treg represents a small percentage of T-cells, and T-cells accounts for a small percentage in the total tumor sample, so including Treg into the regression invariably gives noisy estimations severely driven by other more abundant cell types. In our study, we finalized the 6 immune cell types that are clinically informative yet linearly separable. Across all our inference, we rarely observed strong negative correlations between cell types as observed in CIBERSORT, indicating that our results do not suffer from statistical co-linearity.

**Supplementary Figure Legends**

**Supplementary Figure 1. Distribution of tumor purity across 23 TCGA cancer types.** Tumor purity for each TCGA tumor sample was estimated using DNA SNP array data and R package *CHAT*. Each box in the figure spans 25^th^ to 75^th^ percentile of the data.

**Supplementary Figure 2. Examples of genes with expression levels negatively correlated with tumor purity.** We selected 3 genes, GZMA, CD8A and CTLA4, which are established immune-related genes and displayed their correlations with tumor purity in TCGA kidney renal clear cell carcinoma (KIRC). In all three cases, purity negatively correlated with the gene expression level, indicating that genes were highly expressed in the tumor microenvironment rather than in the malignant cells.

**Supplementary Figure 3. Pathological and computational validation of *in silico* tumor-infiltrating immune cell estimations.** Neutrophil levels were estimated using H&E stained slides and labeled with low, medium and high in BLCA (n=404) (**A**). P values were calculated using Wilcoxon rank sum test. Pathological estimations align well with our computational predictions. Featured samples with low or high estimations were predicted to have abundance of 0.043 and 0.31 respectively in BLCA (**B**), which represent the 2^nd^ and 98^th^ percentiles respectively. Neutrophils have distinctive polymorphonuclear structure under the microscope, as indicated by the green arrow. **C**. DNA-methylation based total leukocyte levels were available in 11 cancers and were compared with the abundance of six immune cell types. Asterisks indicate significance level of 1% FDR. Most of the correlations are strongly positive. Lower correlations are likely due to lower actual infiltrating levels. Neutrophil and dendritic cells overall have the highest correlations, suggest that they are potentially the most abundant of all six infiltrating cell types. **D**. Heatmap displays the Pearson's correlation between estimated immune cell abundance and true values for each of the 23 cancer types. Correlations of 4 comparisons marked with 'X' were below 0.2 and excluded from the downstream analysis.

**Supplementary Figure 4. Impact of viral infection on tumor infiltrating immune cells in HNSC.** Lymphocytes infiltration is affected by virus infection in HNSC, with elevated levels in human papillomavirus positive (HPV+) tumors compared with HPV- tumors. P values were calculated using Wilcoxon rank sum test.

**Supplementary Figure 5. Association of immune infiltration levels and computationally estimated neoantigen load.** Same analysis as Figure 4A was performed using in silico inferred neoantigen counts. Curves span 5^th^ to 95^th^ percentile of the neoantigen count variable and associations with p≤0.1 were displayed, same as in a previous study [[5](#_ENREF_5)]. * indicates significance at FDR≤0.2.

**Supplementary Figure 6. Association of immune infiltration with putative chemokine and receptors.** Expression origin of the chemokine (**A**) and receptors (**B**) was determined by correlation with tumor purity. Positive association suggests cancer cell origin. The abundance of CD8 T-cell and macrophage was compared to the expression of chemokine (**C**) and their receptors (**D**) using partial Spearman's correlation corrected for purity. Multiple test correction was performed in each panel individually and solid entries indicate events significant at 1% FDR.

**Supplementary Figure 7. Additional analysis of immune cell association with cancer vaccine targets.** *SPAG5* (red arrow) significantly associates with CD8 T-cell abundance in prostate, brain, colon, breast and head and neck cancers. Same analysis as in Figure 5 was applied and asterisks indicate events significant at 15% FDR.

**Supplementary Figure 8. Frequency of cancer-specific genes with significant associations to CD8 T cell infiltration levels in multiple cancer types.** For each cancer, same analysis as in Results section **Implications to Cancer Immunotherapy Targets** was performed on cancer-specific genes. Genes significant at FDR≤0.01 were selected and ranked based on their number of occurrence in multiple cancers.

**Supplementary Figure 9. Analysis of inhibitory receptors and ligands.** **A-B**. Associations between CD8 T-cell (**A**) and macrophage (**B**) infiltration and inhibitory molecule expression levels in all cancer types. **C**. Expression origin of putative inhibitory ligands of cytotoxic T cells was determined by correlation with tumor purity. Positive association suggests cancer cell origin. *B7-H3/4* show positive associations with purity in selected cancers. Asterisks indicate significance level of 1% FDR.

**Supplementary Figure 10. Immunohistochemistry analysis on additional TCGA KIRC samples.** Two additional TCGA KIRC samples were analyzed by IHC to support the observations in Figure 6C-E. Samples with high *TIM3* expression showed both low (**A**) and high (**B**) CD8 T cell infiltration. Upper and lower panels are synchronized to visualize co-expression of *TIM3* and *CD8* in the infiltrating T cells. Although synchronized, the panels were not identical because they represented different but closely located layers of the tissue.

**Supplementary Table Legends**

**Supplementary Table 1**. Immune related genes with negative correlation to tumor purity for each cancer type.

**Supplementary Table 2**. Estimated abundance of six immune compartments in the tumor microenvironment across 10,008 TCGA samples.

**Supplementary Table 3**. Comparison of CD8 T cell clinical associations from this study and Fridman et al., 2012 [[6](#_ENREF_6)]

**Supplementary Table 4**. Predicted cancer/testis antigens with significant association to immune cell levels. Statistical tests and significance level estimations were described in Methods.

**Supplementary Table 5**. List genes with cancer-specific expression that are positively associated with immune cell infiltration.

**Supplementary Table 6**. Correlations of immune cell abundance in the tumor microenvironment predicted by TIMER and CIBERSORT. Strong negative correlations potentially indicate statistical co-linearity.

**Supplementary Table 7**. List of samples used as reference immune expression pattern from Human Primary Cell Atlas [[7](#_ENREF_7)].

**Supplementary Table 8**. Complete documentation of statistical results in the study, including Cox regression (Figure 2-3), neoantigen associations (Figure 4), tumor recurrence association with CD8 T cell (Figure 3), correlation of immune cells with inhibitory receptor/ligands (Figure 4, Figure S9). Statistical tests and significance level estimations were described in Methods.

**References**

1. Nelson BH: **CD20+ B cells: the other tumor-infiltrating lymphocytes.** *J Immunol* 2010, **185:**4977-4982.

2. Koch M, Beckhove P, Op den Winkel J, Autenrieth D, Wagner P, Nummer D, Specht S, Antolovic D, Galindo L, Schmitz-Winnenthal FH, Schirrmacher V, Buchler MW, Weitz J: **Tumor infiltrating T lymphocytes in colorectal cancer: Tumor-selective activation and cytotoxic activity in situ.** *Ann Surg* 2006, **244:**986-992; discussion 992-983.

3. Newman AM, Liu CL, Green MR, Gentles AJ, Feng W, Xu Y, Hoang CD, Diehn M, Alizadeh AA: **Robust enumeration of cell subsets from tissue expression profiles.** *Nat Methods* 2015, **12:**453-457.

4. Nagorsen D, Voigt S, Berg E, Stein H, Thiel E, Loddenkemper C: **Tumor-infiltrating macrophages and dendritic cells in human colorectal cancer: relation to local regulatory T cells, systemic T-cell response against tumor-associated antigens and survival.** *J Transl Med* 2007, **5:**62.

5. Rooney MS, Shukla SA, Wu CJ, Getz G, Hacohen N: **Molecular and genetic properties of tumors associated with local immune cytolytic activity.** *Cell* 2015, **160:**48-61.

6. Fridman WH, Pages F, Sautes-Fridman C, Galon J: **The immune contexture in human tumours: impact on clinical outcome.** *Nat Rev Cancer* 2012, **12:**298-306.

7. Mabbott NA, Baillie JK, Brown H, Freeman TC, Hume DA: **An expression atlas of human primary cells: inference of gene function from coexpression networks.** *BMC Genomics* 2013, **14:**632.
